# Characterization of volatiles in flowers from four *Rosa chinensis* cultivars by HS-SPME-GC × GC-QTOFMS

**DOI:** 10.3389/fpls.2023.1060747

**Published:** 2023-05-08

**Authors:** Wenxuan Quan, Jing Jin, Chenyu Qian, Chaochan Li, Hongying Zhou

**Affiliations:** ^1^ Guizhou Provincial Key Laboratory for Information Systems of Mountainous Areas and Protection of Ecological Environment, Guizhou Normal University, Guiyang, China; ^2^ Guizhou Botanical Garden, Guizhou Academy of Sciences, Guiyang, China

**Keywords:** *Rosa chinensis*, metabolomics, volatiles, two-dimensional gas chromatography, quadrupole time-of-flight mass spectrometry

## Abstract

Rosa chinensis cultivars with volatile aromas are important resources in the perfume industry. The four rose cultivars introduced to Guizhou province are rich in volatile substances. In this study, volatiles from four Rosa chinensis cultivars were extracted using headspace-solid phase microextraction (HS-SPME), and analyzed with two-dimensional gas chromatography quadrupole time of flight mass spectrometry (GC × GC-QTOFMS). A total of 122 volatiles were identified; the main compounds in these samples were benzyl alcohol, phenylethyl alcohol, citronellol, beta-myrcene and limonene. A total of 68, 78, 71, and 56 volatile compounds were identified in Rosa ‘Blue River’ (RBR), Rosa ‘Crimson Glory’ (RCG), Rosa ‘Pink Panther’ (RPP), and Rosa ‘Funkuhr’ (RF) samples, respectively. The total volatile contents were in the following order: RBR > RCG > RPP > RF. Four cultivars exhibited similar volatility profiles, with alcohols, alkanes, and esters as the major chemical groups, followed by aldehydes, aromatic hydrocarbons, ketones, benzene, and other compounds. Alcohols and aldehydes were quantitatively the two most abundant chemical groups that included the highest number and highest content of compounds. Different cultivars have different aromas, and RCG had high contents of phenyl acetate, rose oxide, trans-rose oxide, phenylethyl alcohol and 1,3,5-trimethoxybenzene, characterized by floral and rose descriptors. RBR contained a high content of phenylethyl alcohol, and RF contained a high content of 3,5-dimethoxytoluene. Hierarchical cluster analysis (HCA) of all volatiles showed that the three cultivars (RCG, RPP, and RF) had similar volatile characteristics and were significantly different from RBR. Differential metabolites among cultivars were screened based on the OPLS-DA model, and there were six main enriched pathways of differential metabolites: biosynthesis of secondary metabolites, monoterpenoid biosynthesis, metabolic pathways, limonene and pinene degradation, sesquiterpenoid and triterpenoid biosynthesis, and alpha-linolenic acid metabolism. The biosynthesis of secondary metabolites is the most differential metabolic pathway.

## 1 Introduction

Chinese rose (*Rosa chinensis* Jacq.) originated in China and is widely cultivated in China, ancient Egypt, and Greece. It is a well-known ornamental plant with broad applications in the cosmetic and food industries, such as essential oils, spices and natural pigment materials (Qing et al., 2012; Sun et al., 2019; Chen et al., 2021; Cui et al., 2022). Chinese rose is rich in flavonoids and has been used as a raw material in commercial foods and drugs (Cai et al., 2005), and its flowers are commonly used in traditional Chinese medicine. It has long been used as an element in traditional Chinese medicine (Cai et al., 2005; Qing et al., 2012; Luo et al., 2020). Furthermore, several Chinese rose cultivars have been cultivated in China for more than 1,300 years to produce perfumes. Some cultivars are used as edible flowers and as ingredients in the food, brewing and pharmaceutical industries to improve the quality of products (Luo et al., 2020). The volatile compounds found in *Rosa* flowers play an important role in aroma and determine the quality of fragrance. It remains unclear which volatiles are similar, which are unique, and what the volatile profile characteristics are among different rose cultivars. Therefore, investigating volatile compounds improves the utilization of the aromatic characteristics of *Rosa*. Due to the lack of literature on the volatile chemical components of *Rosa* flowers, it is necessary to study and identify specific cultivars to utilize these rich *Rosa* resources.

A single cultivar is not representative, as significant differences in volatile composition occur among cultivars, and different constituent substances may affect the fragrance, both individually and synergistically. Flower fragrance is an important characteristic of Chinese rose, and many cultivars are classified according to their aromatic components (Du et al., 2019; Gil et al., 2020; Zhou et al., 2020). In this study, we chose the *Rosa* ‘Funkuhr’, *Rosa* ‘Pink Panther’, *Rosa* ‘Crimson Glory’ and *Rosa* ‘Blue River’ cultivars of Chinese rose planted at the Guizhou Botanical Garden. *R.* ‘Crimson Glory’ and *R.* ‘Blue River’ possess highly fragrant flowers, while *R.* ‘Pink Panther’ and *R.* ‘Funkuhr’ present mainly moderate and minimal fragrances, respectively. In addition, volatiles in *Rosa* flowers reflect the fragrance more effectively, we used fresh petals as research materials. Headspace solid-phase microextraction (HS-SPME) is a convenient alternative to more conventional methods for extracting volatiles and semivolatiles from different sources, as it extracts volatiles without affecting the extracted chemicals (Özgenç et al., 2017; Al-Dalali et al., 2019). HS-SPME has been successfully used to extract volatiles from plants, such as *Olea europaea*, *Trigonellafoenum-graecum* (Baccouri et al., 2022; Rajhi et al., 2022), *Rosa rugose* and *Rosa roxburghii* (Sheng et al., 2021; Huang et al., 2022). The two-dimensional gas chromatography quadrupole time-of-flight mass spectrometry (GC × GC-QTOFMS) methodology has been used to profile the volatile organic compounds in *Malus domestica*, *Rosa hybrida* and *Cucumis sativus* (Risticevic et al., 2012; Pico et al., 2018; Amrapali et al., 2020; Jo et al., 2022).

This study combines the methods of univariate statistical analysis and multivariate statistical analysis, accurately mining the differential volatiles among four cultivars of Chinese rose. Investigated characteristics of volatile compounds and differential metabolites in different cultivars can provide basic data for aroma characteristics, as well as provide reference for the development of essential oils and medicinal values of specific cultivars of Chinese rose.

## 2 Materials and methods

### 2.1 Materials and samples

**Figure 1:**
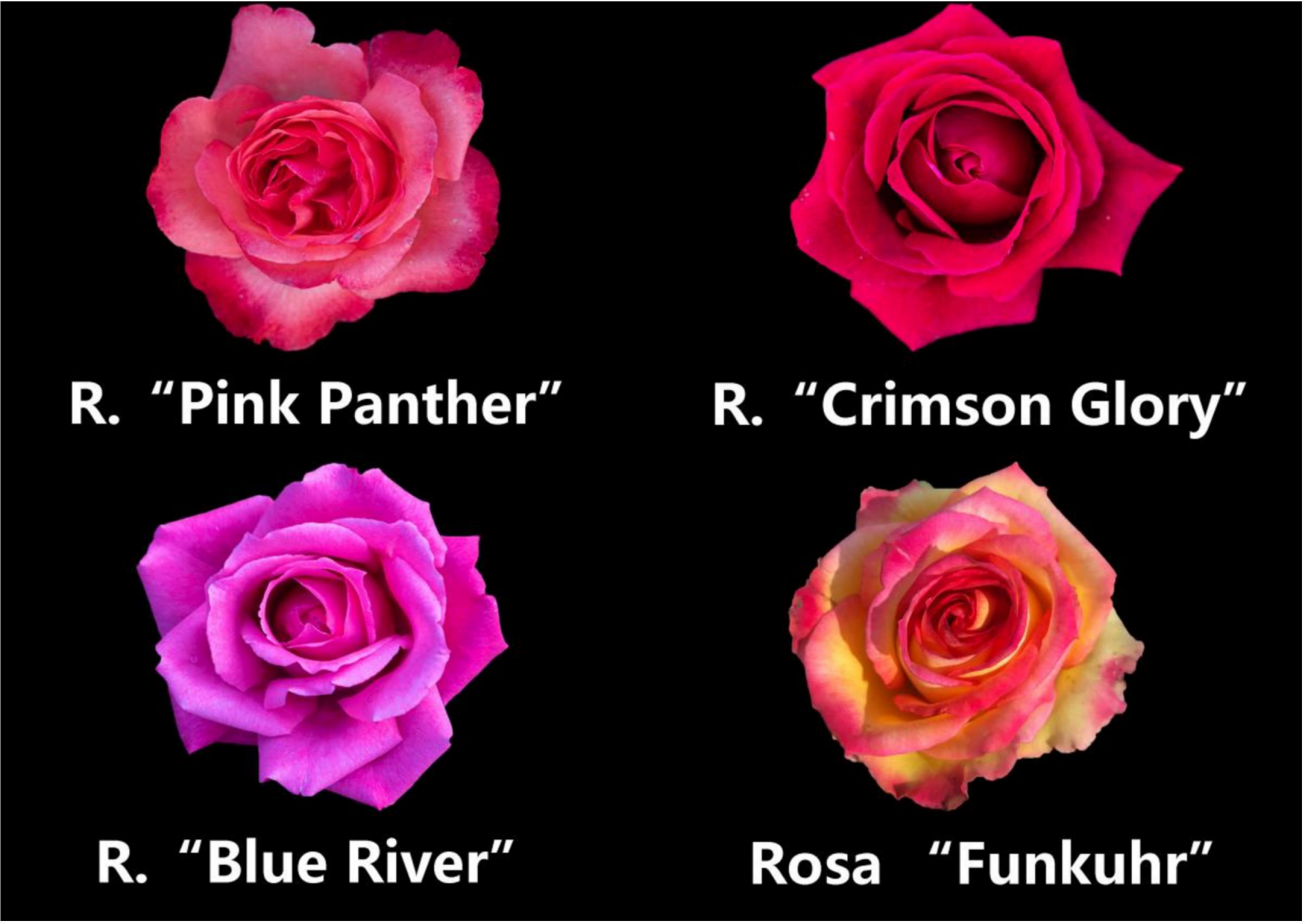
*Rosa* samples from the four cultivars.

The Chinese rose cultivars have different colors and fragrances, and the four cultivars selected in this study had large color differences. Four cultivars (*Rosa* ‘Funkuhr’, *R.* ‘Pink Panther’, *R.* ‘Crimson Glory’, and *R.* ‘Blue River’, denoted RF, RPP, RCG and RBR, respectively) were generously provided by the experimental field of Guizhou Botanical Garden and harvested at E 106° 40’–27”; N 26° 11’ 39”, located in Guiyang City, China (**Figure 1**). The flowers were harvested before petal opening and transferred to the laboratory. The samples were powdered following the method described in literatures (Qian et al., 2019; Gil et al., 2020). Methanol was purchased from Sigma−Aldrich Fluka (Buchs, Switzerland). Methyl hexadecanoate (internal standard; purity ≥ 97.5%) was obtained from CATO (Portland, OR, USA) and stored at 4°C until use. Commercially available SPME coating fibers, including 85 µm carboxen/polydimethylsiloxane (CAR/PDMS), 50/30 µm divinylbenzene/carboxen/polydimethylsiloxane (DVB/CAR/PDMS), 65 µm polydimethylsiloxane/divinylbenzene (PDMS/DVB), and 60 µm carbowax-polyethylene glycol (PEG), were purchased from Supelco (Bellefonte, PA, USA).

### 2.2 Optimization of SPME conditions

Four SPME fibers were tested, including PDMS/DVB, DVB/CAR/PDMS, PDMS/CAR, and PEG. To ensure no interference, all fibers were thermally cleaned at the condition temperature after each set of measurements. Additionally, each type of coating was tested in triplicate during evaluation of the SPME method. DVB/CAR/PDMS fibers yielded the highest peak areas for most volatile compounds, such as limonene, 3,5-dimethoxy toluene, and citronellol. In contrast, PEG yielded the lowest peak areas for the extracted volatile compounds, suggesting that only a small fraction of highly polar compounds, such as alcohols and benzene, were extracted. Between PDMS/DVB and CAR/PDMS, CAR/PDMS yielded higher peak areas for low molecular weight volatiles, such as (*E*)-2-hexanal, while PDMS/DVB was more suitable for high molecular weight volatiles, such as benzyl alcohol. DVB/CAR/PDMS captured more compounds with a wider range of molecular weights and was selected as the best option to detect volatiles in rose samples (**Figure S1**).

### 2.3 GC×GC-QTOF MS: instrument setup and conditions

GC × GC analysis was performed using gas chromatography (7890B Agilent Technologies, Palo Alto, CA, USA) coupled with high resolution QTOFMS preparation with a PAL RSI 120 autosampler (CTC Technologies). The oven temperature was initially programmed at 50°C (held for 1 min) and then increased to 260°C at a rate of 5°C/min (held for 2 min at the final temperature). The inlet temperature was set to 260°C, and the transfer line temperature was 280°C. Electron impact ionization was employed, with electron energy applied at 70 eV. The ion source temperature was set to 250°C, and the mass range was set to 50–500 m/z in the full-scan acquisition mode. Helium (99.999%) was used as the carrier gas at a constant flow rate of 1.2 mL/min. The combination of an HP-5 MS column of the first dimension (5% phenyl-95% dimethylpolysiloxane, 30 m × 250 μm, 0.25 μm film) and a DB-17 MS column of the second dimension (50% phenyl-50% dimethylpolysiloxane, 1.2 m × 180 μm, 0.18 μm film) was used as the two-dimensional capillary column system (Agilent, Little Falls, DE, USA).

For each sample, a 100 mg sample and 2 μL of internal standard (IS: methyl hexadecanoate, 100 ppm) were transferred to a 20 mL glass vial. Volatile compounds were equilibrated for 20 min at 60°C in a glass vial and then extracted for 15 min with SPME fibers (DVB/CAR/PDMS). The fibers were then immediately inserted into the GC injection port to desorb the volatile compounds at 260°C for 3 min in splitless mode. Quality control (QC) samples were composed of 300 mg of mixed petals per cultivar, and QC samples were used to optimize the HS-SPME method.

GC × GC data were analyzed using Canvas dedicated GC × GC data processing software (J&X Technologies, version v1.6.0).

### 2.4 Compound identification and method performance parameters

Tentative identification of compounds in the rose samples was primarily performed with matching factor screening, followed by comparison of MS spectra and experimental retention index (RI_exp_) based on the NIST 17 library. The minimum MS match factor was set at 750 for both the direct match factor (DMF) and reverse match factor (RMF) based on previous studies (Cordero et al., 2019; Stilo et al., 2019; Cialiè Rosso et al., 2021). **Table S1** shows high similarities (up to 935) in the mass spectra of the identified compounds, supporting the reliability of the results. The experimental 1D retention indices of each compound were calculated using the n-alkane series (C_8_–C_25_). In GC × GC analysis, the relatively large deviation that exists between RI_exp_ and the retention index of the literature (RI_lit_) due to the phasing of modulation was considered reasonable (Mondello et al., 2015). Therefore, relatively large RI windows were applied for compound screening purposes, and compounds with differences of more than 40 between experimental 1D retention indices and the literature were excluded from this study. Additionally, GC × GC coupled with high-resolution MS allowed for coelution to be minimized and for identification based on molecular ions to be more accurate (Schwanz et al., 2019). Here, an exact mass analysis with a mass accuracy of < 5 ppm was achieved.

Thirteen potential major volatiles and an internal standard (IS: methyl hexadecanoate) were chosen to calculate the relative standard deviations (RSDs) of retention times to confirm the stability of the GC × GC/QTOFMS methodology through previous literature studies (Zhou et al., 2020). In total, five replicate analyses were performed for each cultivar. The average RSDs for the retention times of the thirteen selected compounds were 0.02% and 0.37% for the 1D and 2D retention times, respectively (**Table 1**). These RSD values showed that the HS-SPME-GC × GC/QTOFMS method was sufficiently stable and precise.

**Table 1:** Repeatability data for the retention times of selected volatiles by HS-SPME-GC×GC/QTOF-MS.

Constituents	1^st^ dimension		2^nd^ dimension	
	*t*_R_^a^ (min)	%RSD^b^ (n=20)	*t*R^a^ (sec)	%RSD^b^ (n=20)
Hexanal	5.19	0.00	1.68	0.70
2-Hexenal	6.29	0.08	2.27	0.57
beta-Myrcene	9.69	0.04	1.83	0.76
Benzaldehyde	8.99	0.00	3.99	0.24
Limonene	10.79	0.00	2.09	0.13
Benzyl alcohol	10.99	0.04	4.21	0.12
Benzeneacetaldehyde	11.19	0.00	4.59	0.20
beta-Ocimene	11.29	0.00	2.06	0.63
Phenylethyl alcohol	13.29	0.00	4.42	0.41
D-Citronellol	16.29	0.03	2.70	0.40
Geraniol	17.09	0.00	2.96	0.18
3,5-Dimethoxytoluene	17.39	0.02	4.45	0.10
1,3,5-trimethoxybenzene	21.19	0.00	5.35	0.18
IS^c^	32.79	0.00	2.39	0.57

^a^1D and 2D retention time; ^b^relative standard deviation; ^n^number of analyses; ^c^Internal standard.

The categories of compounds isolated from the rose samples and the mean values of three extractions and their standard errors are shown. Furthermore, the aroma descriptors of most compounds in the literature are also listed. Their relative content was calculated using the respective content based on the proportion of the peak areas of the compounds relative to the internal standard (**Table S1**).

### 2.5 Data analysis

Univariate statistical analysis was performed using R software. Statistical significance was assessed by one-way analysis of variance (ANOVA) followed by the least significant difference (LSD) test, and a p value < 0.05 was considered significant.

Differential metabolites selected: Combining the methods of univariate statistical analysis and multivariate statistical analysis, we eventually accurately extracted the differential metabolites. Multivariate statistical analysis methods include principal component analysis (PCA), hierarchical cluster analysis (HCA), and orthogonal partial least squares discriminant analysis (OPLS-DA). The significantly regulated metabolites between these groups were determined by VIP value > 1 and p value < 0.05 (Zhang et al., 2022). VIP values were extracted from the OPLS-DA results, which also contained score plots and permutation plots generated using the R package MetaboAnalystR (The R Foundation for Statistical Computing, Vienna, Austria).

Unsupervised principal component analysis (PCA) was performed using prcomp in R version 3.5.1. Data were scaled unit variance before unsupervised PCA (Ren et al., 2016; Quan et al., 2023). The hierarchical cluster analysis (HCA) results of the samples and metabolites are presented as heatmaps with dendrograms. The HCA of the accumulation pattern of metabolites among different samples was carried out using the R package ComplexHeatmap version 2.8.0 (Quan et al., 2023).

The identified metabolites were annotated using the KEGG compound database, and the annotated metabolites were assigned to the KEGG pathway database (http://www.kegg.jp/kegg/pathway.html). Pathways with significantly regulated metabolites were fed into MSEA (metabolite set enrichment analysis), and their significance was determined by hypergeometric test p values. All analyses were performed with the R software package (Cao et al., 2022).

## 3 Results

### 3.1 Analysis and identification of volatile compositions in the four cultivars

**Figure 2:**
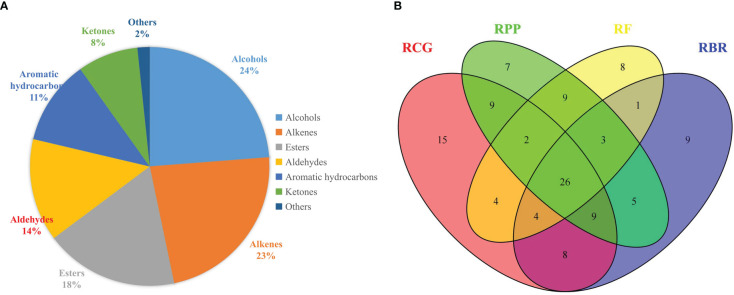
Functional groups of volatiles **(A)** and Venn diagram for the number of volatile compounds **(B)**.

A total of 122 volatile compounds were identified in the flowers of four *Rosa* cultivars, and these volatiles are shown in **Table S2**. Volatiles in the four cultivars were diverse, including representatives of 7 functional groups: alcohols, alkanes, esters, aldehydes, aromatic hydrocarbons, ketones, benzenes, and others. Alcohols, alkanes, and esters were the main functional groups; the other minor compounds, such as aldehydes, aromatic hydrocarbons, ketones, and others, balanced the composition of the specific fragrances of the *Rosa* flowers (**Figure 2A**, **Table S2**).

A total of 68, 78, 71, and 56 volatile compounds were detected and identified in the RBR, RCG, RPP, and RF samples, respectively. Additionally, the content of volatiles varied widely among the *Rosa* samples. In total, 15, 9, 7 and 8 unique components were found in the RCG, RBR, RPP and RF samples, respectively (**Figure 2B**). RBR had the highest content of total volatiles, followed by RCG, RPP, and RF, and the total volatile content in FBR was 1,869.54 μg/g, followed by RCG at 1,843.99 μg/g. The highest alcohol content was observed in RBR and RCG, and the highest aldehyde content was detected in RPP and RF (**Table S2**).

### 3.2 The main chemical groups of volatile compounds

Alcohols were quantitatively the most abundant chemical group that included the highest number and content of compounds, particularly in RBR and RCG. Alcohols had the highest content with a rose aroma among all of the chemical groups. The most common alcohols in the flowers of RBR and RCG were benzyl alcohol, phenylethyl alcohol, citronellol and geraniol; the typical scent of rose flowers is attributed to these volatiles.

Aldehydes were another major group of total volatile compounds, and a similar number of aldehydes were detected in the four cultivars. Hexanal, 2-hexenal, and benzeneacetaldehyde were the most abundant aldehydes. 2-hexenal was dominant in all rosa cultivars, accounting for the highest content in RBR and RF; however, the total content of volatile compounds in RF was significantly lower than that in RCG. Benzeneacetaldehyde has a ‘honey’ aroma, and this compound was detected in all samples. Other important aldehydes that contribute to the fragrance of *Rosa* species include benzaldehyde (sweet, sugar) and decanal (sweet, floral). Two unique compounds, (*E,Z*)-2,4-heptadienal and 2-phenyl-2-butenal, were detected only in RF and RCG, respectively, and could have potentially contributed to the ‘green’ and ‘sweet’ aromatic attributes (**Table S2**).

Alkenes were present in large quantities in RBR and RCG, followed by RPP and RF. Beta-myrcene, *d*-limonene, and trans-ocimen were the dominant compounds among the alkenes. In contrast, alpha-phellandrene and alpha-muurolene were present with low contents (<0.5 μg/g) in RF. Furthermore, most alkenes were mainly associated with the ‘woody’ descriptor, including caryophyllene, alpha-muurolene, and beta-cadinene.

Twenty-two esters were identified in *Rosa* flowers, and 2-phenethyl acetate was the ester with the highest content in RCG and has been characterized by its floral and rose descriptors. Additionally, several esters presented a rose fragrance, such as geranyl formate (fresh, rose), citronellol acetate (floral, rose), and nerol acetate (floral, rose). In contrast, aromatic hydrocarbons had fewer compounds but higher contents (**Table S2**). RF and RBR contained a high content of 3,5-dimethoxytoluene, while RCG contained a high content of 1,3,5-trimethoxybenzene (>50 μg/g).

### 3.3 Principal component analysis and Pearson correlation coefficients of four cultivars

**Figure 3:**
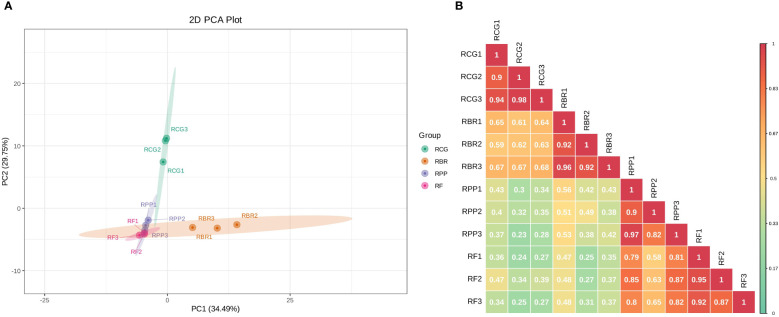
2D plot of principal component analysis **(A)** and correlation diagram among samples **(B)**. **(A)** the percentage represents the interpretation rate of the principal component in the dataset; **(B)** Different colors represent different Pearson correlation coefficients. The redder the color, the stronger the correlation, and the greener the color, the worse the correlation.

Principal component analysis by covariance matrix was performed on 122 volatile components of four rosa cultivars, and principal component 1 (PC1) and PC2 explained 34.49% and 29.75% of the variation, respectively. In PC1, RF and RPP showed low loads, while RBR showed high loads. In PC2, RF, RBR and RPP showed low loads, while RCG exhibited high loads (**Figure 3A**). The PCA of the samples revealed an obvious separation between RCG and the other three cultivars; there was no significant difference in the three samples within each cultivar.

Pearson’s correlation coefficient (r) was used as an evaluation index for biological repetitive correlation. The closer the r-value is to 1, the stronger the correlation between two duplicate samples. The results showed that there was a strong correlation among samples within four cultivars, with r-values greater than 0.82. The correlation between groups showed that RCG and RBR had a strong correlation, while RPP and RF had a strong correlation (**Figure 3B**).

### 3.4 Hierarchical cluster analysis of four cultivars

**Figure 4:**
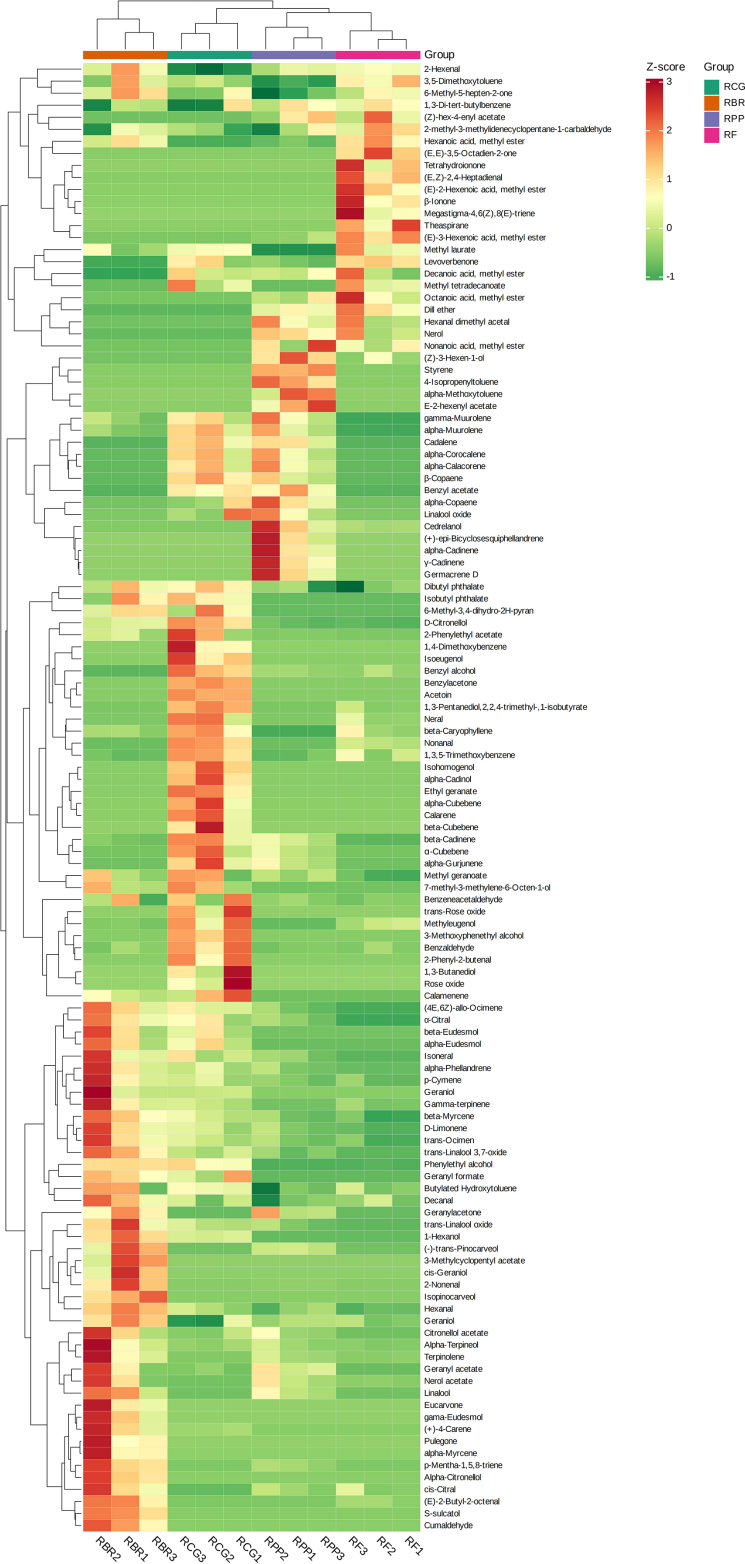
Clustering heatmap based on all volatile contents. Note: the horizontal line represents the sample name, the vertical line represents the volatiles, and the different colors are relative contents (red represents high content, green represents low content).

The HCA revealed that the high relative content and unique volatiles in each sample play an important role in clustering. For example, (+)-4-carene, alpha-phellandrene, alpha-terpineol, cis-citral, citronellol acetate, isoneral, nerol acetate, terpinolene, etc., were higher in RBR than in the other three cultivars. The contents of 2-phenylethyl acetate and beta-cubebene, etc., were higher in the RCG group than in the other three cultivars, the contents of (*Z*)-3-hexen-1-ol, alpha-copaene, alpha-muurolene, gamma-muurolene, cedrelanol, etc., were higher in the RPP group than in the other three cultivars, the contents of (*E*)-3-hexenoic acid, methyl ester, decanoic acid, methyl ester, dill ether, octanoic acid, methyl ester, etc., were higher in RCG than in the other three cultivars (**Figure 4**).

### 3.5 Orthogonal partial least squares discriminant analysis

**Figure 5:**
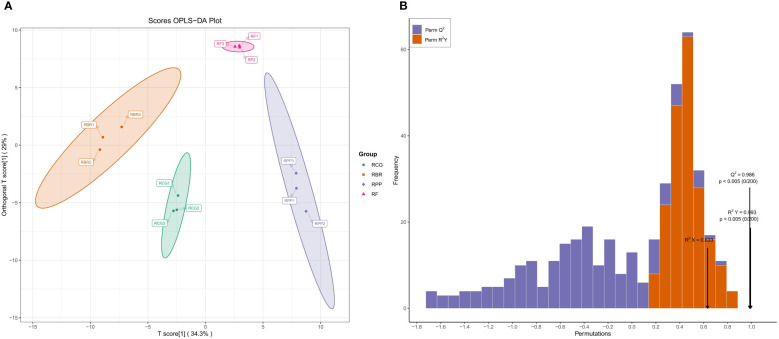
OPLS-DA score and verification based on all volatiles. **(A)** Scores OPLS-DA; **(B)** Validation of OPLS-DA model.

The difference between the groups can be seen in the abscissa direction, where the difference within RBR and RCG is small, while the difference within the other groups is large. Differences within the group can be seen in the vertical coordinate direction, with relatively large differences within the RPP, RBR and RCG groups (**Figure 5A**).

OPLS-DA model validation: R^2^X, R^2^Y, and Q^2^ represent the interpretation rate of the X and Y matrices and the prediction ability of the model, which are 63.3%, 99.3%, and 98.6%, respectively. This model conducts 200 random permutation and combination experiments on data, and the p<0.005 of Q^2^ indicates that the prediction ability of at most one random grouping model in this permutation test is superior to this OPLS-DA model. The p<0.005 for R^2^Y indicates that at most one random grouping model in this permutation test has a better interpretation rate for the Y matrix than this OPLS-DA model (**Figure 5B**).

### 3.6 Screening of differential metabolites among four cultivars

**Figure 6:**
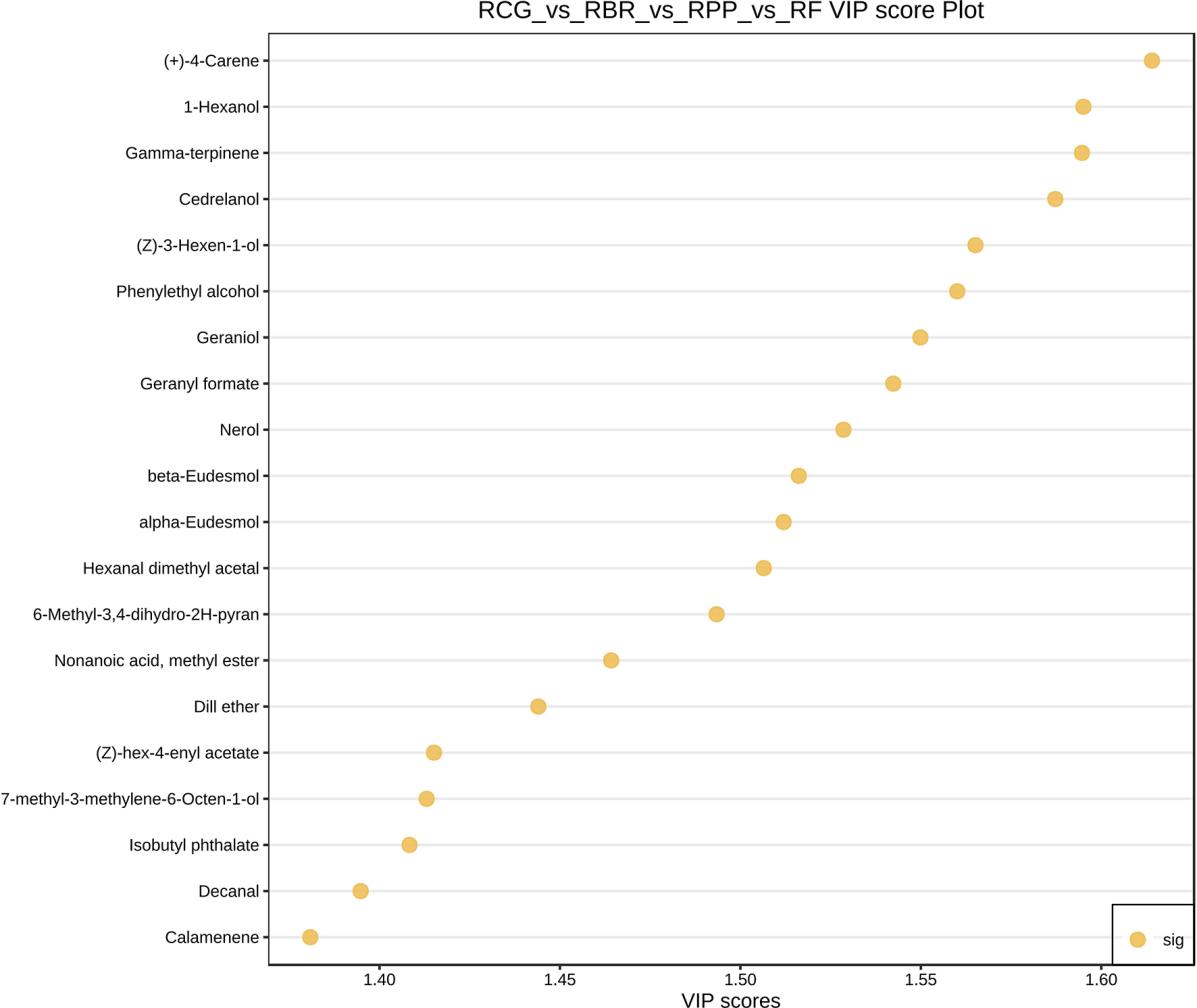
Variable importance in projection (VIP) score chart of differential metabolites.

Based on the variable importance in projection (VIP) obtained from the OPLS-DA model, metabolites with differences between different cultivars were initially screened. The top 20 metabolites selected for VIP values in the OPLS-DA model are shown in **Figure 6**.

**Figure 7:**
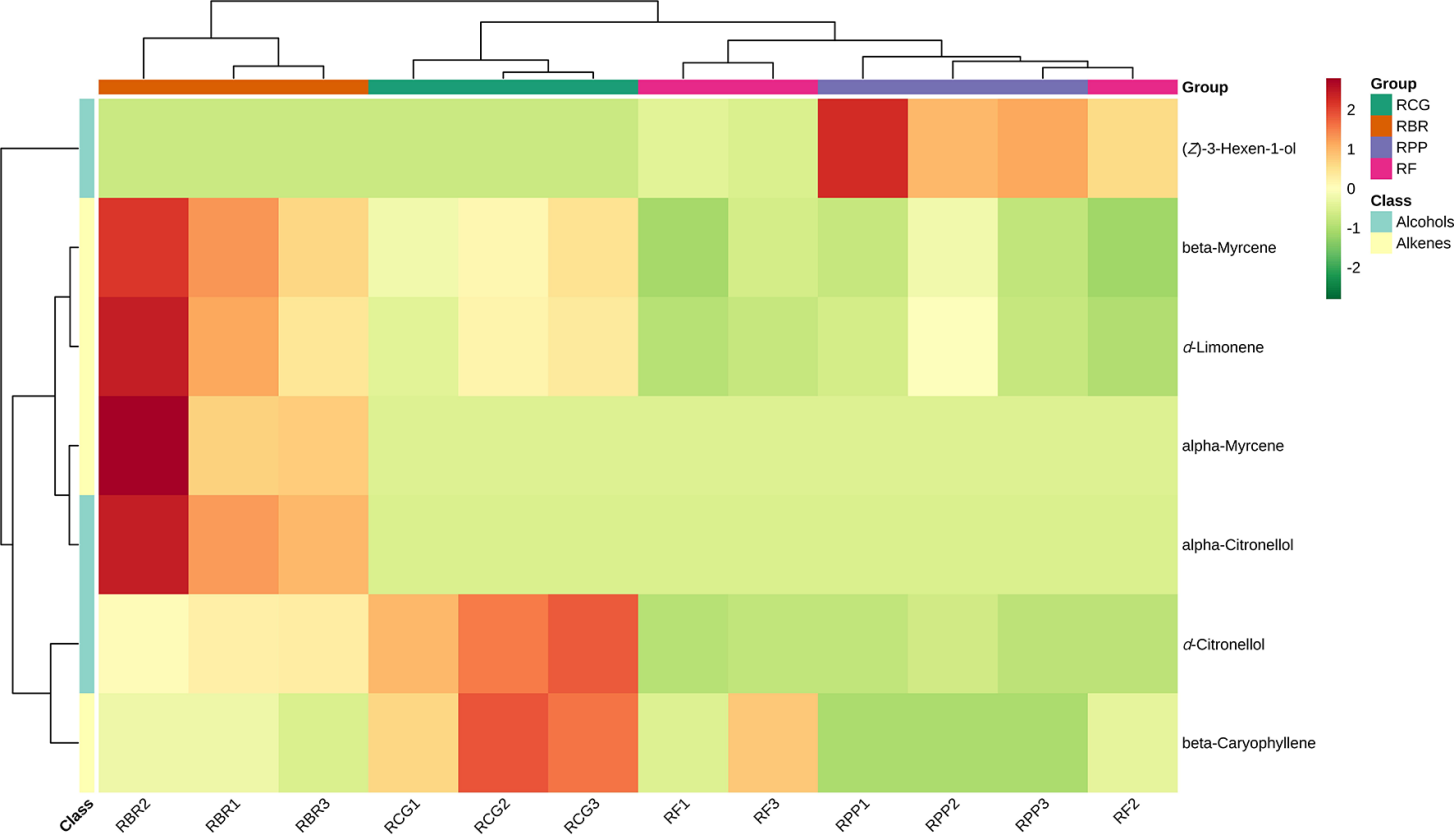
Clustering heatmap KEGG pathway of differential metabolites. Note: Red represents high content, and green represents low content.

Using KEGG annotation information for differential metabolites identified according to the screening criteria, 5 significantly enriched KEGG metabolic pathways were selected, and hierarchical cluster analysis of all differential metabolites in these pathways was performed. HCA of the abundance of 7 differential compounds was also performed to categorize the cultivars and resulted in two groups (**Figure 7**). The differential metabolites in RBR are different from the other three cultivars, among which beta myrcene, d-limonene, alpha myrcene, and alpha-citronellol have high content characteristics in RBR. D-citronellol and beta caryophyllene had high contents in the RCG, and (*Z*)-3-hexen-1-ol had high contents in RPP (**Figure 7**).

**Figure 8:**
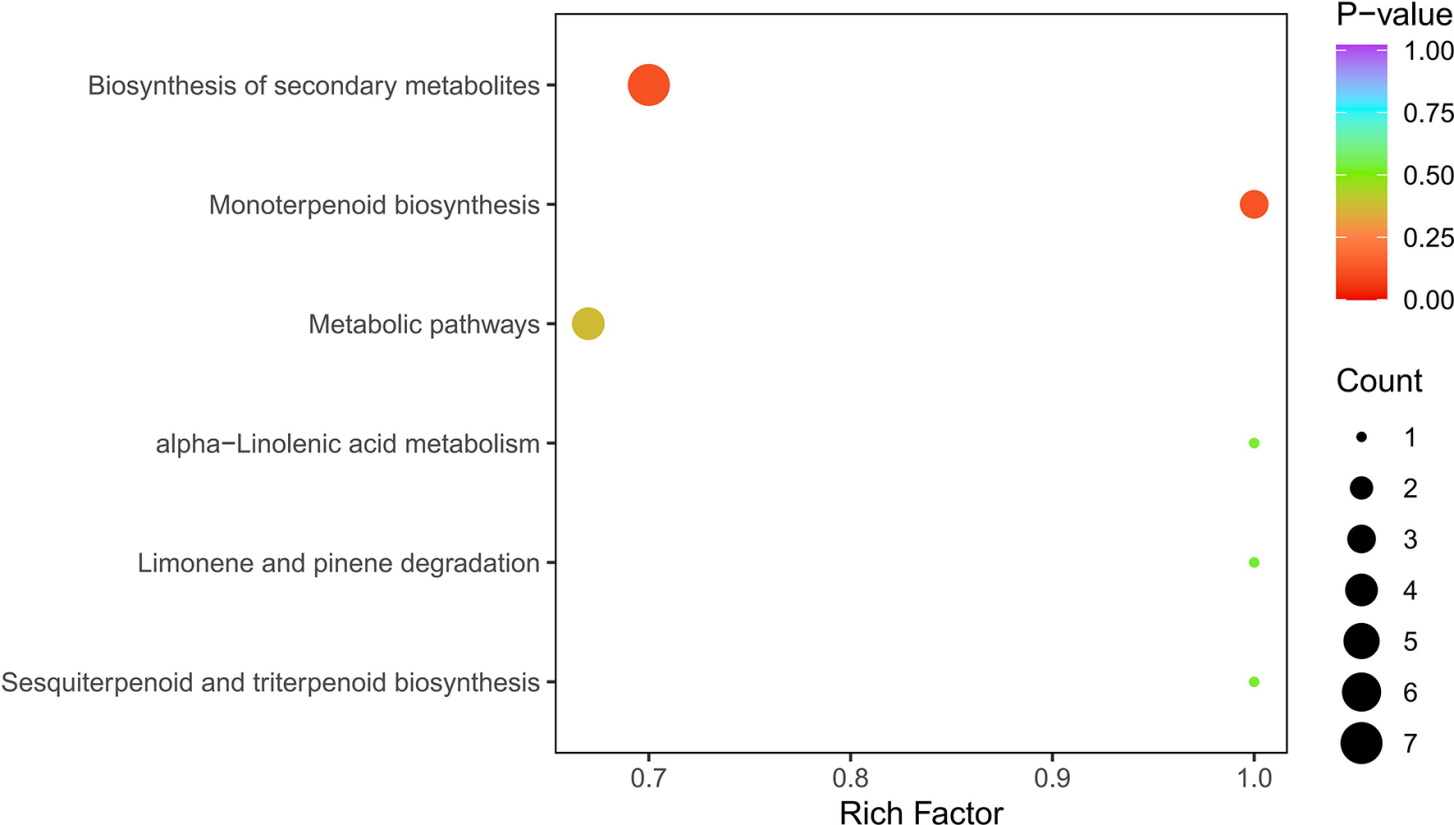
KEGG enrichment of differential metabolites. Note: The abscissa represents the rich factor corresponding to each pathway, and the ordinate is the pathway name.

Based on the metabolite results, a KEGG pathway enrichment analysis was carried out, and the most significantly enriched pathways were biosynthesis of secondary metabolites (7 out of 9), monoterpenoid biosynthesis (3 out of 9), metabolic pathways (4 out of 9), limonene and pinene degradation (1 out of 9), sesquiterpenoid and triterpenoid biosynthesis (1 out of 9), and alpha-linolenic acid metabolism (1 out of 9) (**Figure 8**).

## 4 Discussion

### 4.1 The functional groups and fragrance of rose volatile compounds

The use of a chemical taxonomy of volatile compounds from rose cultivars can help explain variety differences and identify them (Zhou et al., 2020). Volatiles in the four cultivars, including representatives of seven functional groups, alcohols, alkanes and esters, were the main functional groups, probably because these functional groups play key roles in the formation of the rose fragrance (Feng et al., 2010). Some volatile compounds contributed significantly to the fragrance, while others built up the background fragrance, and some trace compounds were the unique fragrance profiles of the Rosa cultivars.

Alcohols were quantitatively the most abundant chemical group, particularly for RBR and RCG. Among all chemical groups, alcohols presented the highest content of compounds with the ‘rose’ aroma. The most prevalent alcohols in *Rosa* flowers are benzyl alcohol, phenylethyl alcohol, citronellol, geraniol, and nerol (Yousefi et al., 2021). The latter three compounds, which compose the rose and floral fragrance (Qin et al., 2013; Nedeltcheva-Antonova et al., 2017; Nunes and Miguel, 2017; Feng et al., 2018), were identified as the major compounds in all samples. Other components, such as alpha-terpineol and linalool oxides, are very important aromatic components that provide a floral and sweet fragrance (Lv et al., 2012). Furthermore, both L-pinocarveol and isopinocarveol were associated with a woody aroma. Although different *Rosa* samples had similar chemical profiles, the volatile content of the two selected compounds (benzyl alcohol and phenylethyl alcoho) demonstrated significant differences among the rose cultivars. The corresponding raw extracted ion chromatograms for benzyl alcohol and phenylethyl alcohol from the rose cultivars are shown in the top and bottom panels, respectively (**Figure S2**). Of particular interest is benzyl alcohol, which had a relatively high content in RCG, RF, and RPP and represents a sweet and floral aroma (Zhu et al., 2016). However, RBR and RCG contained relatively high contents of phenylethyl alcohol, followed by RF and RPP. Combining previous research results, phenylethyl alcohol may be closely associated with the strong rose scent of RBR and RCG due to its high content (Lei et al., 2015; Hirata et al., 2016).

Aldehydes were another main component of total volatiles, and a similar number of aldehydes were detected in all four cultivars. Hexanal, 2-hexenal, and benzeneacetaldehyde were the most abundant aldehydes. In particular, 2-hexenal, which has a green and fruity fragrance (Feng et al., 2019), was dominant in all cultivars. Benzeneacetaldehyde has a ‘honey’ aroma and was detected in all samples. Other important aldehydes that contribute to the fragrance of *Rosa* cultivars include benzaldehyde (sweet, sugar) and decanal (sweet, floral) (Amanpour et al., 2019; Feng et al., 2019). Several volatiles in the alkenea and ketones possessed pleasant fragrances. Alkenes were present in high amounts in RCG and RPP, followed by RBR and RF. Among the alkenes, beta-myrcene, limonene, and beta-ocimene were dominant. They potentially contribute to fragrance ‘fruity’ (beta-myrcene), ‘fresh and citrusy’ (*d*-limonene), or ‘floral’ (beta-ocimene) (An et al., 2019). Furthermore, most alkenes are mainly associated with the ‘woody’ descriptor, including caryophyllene, alpha-muurolene, and beta-cadinene. In particular, rose oxide and trans-rose oxide were found only in RCG, and the two compounds are key flavor components that contribute to the distinctive rose fragrance and may have contributed to the ‘rose’ aromatic attribute in RCG (Nedeltcheva-Antonova et al., 2017; Feng et al., 2018). Ketones include volatile compounds that possess fresh or fruity aromas (Feng et al., 2018; Song et al., 2020); for example, 6-methyl-5-hepten-2-one is the most powerful aromatic compound, characterized by fruity and green attributes (Qin et al., 2013). *Rosa* samples possessed a rich variety of esters, and 2-phenylethyl acetate in RCG had the highest content and was characterized by floral and rose descriptors. Additionally, several esters presented a rose fragrance, such as geranyl formate (fresh, rose), citronellol acetate (floral, rose), and nerol acetate (floral, rose). Most esters were detected in RBR and RCG, and a small amount was detected in RF and RPP. In contrast, aromatic hydrocarbons had a high content; RF and RBR contained a high content of 3,5-dimethoxytoluene, while RCG contained a high content of 1,3,5-trimethoxybenzene. Volatiles in many rose cultivars are dominated by 3,5-dimethoxytoluene and 1,3,5-trimethoxybenzene (Scalliet et al., 2002; Wu et al., 2004; Ren et al., 2016), and these compounds continue to be produced after flower harvest (Zeng et al., 2019). These compounds contributed to the unique fragrance of the *Rosa* cultivars.

Aromatic substances (volatiles) are used as quality assessment parameters for all cultivars, and volatiles are the main components of essential oils, which have a significant economic benefit (Zhou et al., 2020). This study adopted a metabolomics analysis method to screen volatiles, which are also the main substances in rose flowers, such as *d*-citronellol, benzyl alcohol, geraniol, and phenylethyl alcohol, with stable properties and high antioxidant, antidepressant and antibacterial activities (Koksal et al., 2015; Ren et al., 2016). The rose flowers have various important bioactivities and medicinal value (Nayebi et al., 2017; Nunes and Miguel, 2017; Akram et al., 2019).

### 4.2 PCA, HCA, and OPLS-DA for multivariate analysis of volatile compounds

Using multivariate statistical analysis simplifies and reduces the dimensions of high-dimensional and complex data while maximizing the retention of original information and establishing a reliable analytical model to summarize the metabolic spectrum characteristics of the study object (Deng et al., 2021). PCA method is often used to study how to reveal the internal structure of multiple variables through a few principal components, that is, to derive a few principal components from the original variables so that they retain as much information as possible about the original variables (Eriksson et al., 2006). In the four cultivars, the PCA results revealed an obvious separation between RCG and the other three cultivars, indicating that RCG formed unique characteristics of volatile substances during growth and metabolism and was significantly different from other cultivars. Metabolomics data are multidimensional, so combining univariate and multivariate statistical analyses and analyzing data from a multivariate perspective based on data characteristics can accurately analyze differential metabolites. Before conducting a differential analysis, first perform principal component analysis on the grouped samples for difference comparison, and observe the degree of variation between the different groups and within the group samples.

OPLS-DA can maximize intergroup differentiation and facilitate the search for differential metabolites. VIP value of OPLS-DA represents the impact of differences between corresponding metabolites on the classification and identification of each group of samples in the model. It is generally believed that metabolites with VIP > 1 are significantly different (Bi et al., 2022). Based on the fact that volatiles and aroma are important reference indicators for breeding different rose varieties (Zhou et al., 2020; Feng et al., 2022), there are significant differences in the content of alcohols and alkenes among different rose varieties, indicating that breeding of rose varieties mainly involves changing the secondary metabolic pathway to synthesize different levels of secondary metabolites (such as alcohols and alkenes).

### 4.3 Advantages of HS-SPME and GC×GC/QTOFMS detection technology

Headspace analysis is a simple, nondestructive, and solvent-free technology to extract volatile compounds from liquid and solid matrices. This technology has been widely used in the food and spice industry to generate the flavor characteristics of various foods and spices (Kamatou et al., 2019; Rubegeta et al., 2019). Since the introduction of the SPME technique in the late 1980s by Pawlyszin’s group, it has gained much attention in the analysis of a wide spectrum of samples, for example, food, medical, biological, etc. (Mohammadhosseini, 2015a; Mohammadhosseini, 2015b; Bendif et al., 2020; Chizzola and Müllner, 2020). HS-SPME is an analytical technique that combines solid-phase microextraction with headspace injection, SPME fibers have been the most popular headspace method used in recent years (Moniruzzaman et al., 2014; Sybille et al., 2015; Godage and Gionfriddo, 2019).

In the present study, the GC × GC system comprised a long nonpolar 1D column to allow volatility-based separation and a significantly shorter, polar 2D column to allow for polarity-based separation. GC × GC-QTOFMS combined with HS-SPME extraction using an autosampler provided good reproducibility of retention times (Ntlhokwe et al., 2018). This typical combination of two different retention mechanisms achieved better resolution efficiency based on the boiling point and polarity, resulting in the separation and identification of a greater number of compounds. Furthermore, the GC × GC method achieved better overall chromatographic separation, expanding the efficiency of the available separation space. 3,5-dimethoxytoluene and 1,3,5-trimethoxybenzene are the main scent components in Chinese roses (Scalliet et al., 2002); however, 3,5-dimethoxytoluene and phenylethyl alcohol were not found in Chinese rose (Zhou et al., 2020). We used GC × GC-QTOFMS technology to detect an extremely high content of phenylethyl alcohol in the RCG and RBR cultivars, as well as an extremely high content of 3,5-dimethoxytoluene in the RCG, RBR and RF cultivars.

## 5 Conclusion

This study investigated the volatile compounds in four cultivars of Chinese rose and provided strong evidence of the volatile compounds of rose cultivars. A total of 122 volatiles were identified, and the most abundant volatile compounds were alcohols, alkanes, and esters. These volatile compounds determined the special flavors of Chinese rose cultivars. These experimental results revealed that the content of these metabolites varies significantly among different cultivars. KEGG enrichment analysis explained why volatile compounds (secondary metabolites) were different in the four Chinese rose cultivars. Furthermore, more volatiles provide a reference and selection of Chinese rose cultivars for the actual production of rose essential oils, and the main volatile compounds could be a basis for medicinal value. In addition, the SPME fiber assembly divinylbenzene/carboxen/polydimethylsiloxane (DVB/CAR/PDMS) was capable of capturing more compounds with a wider range of molecular weights and was more suitable for detecting volatiles in rose samples. This study analyzed the metabolic enrichment pathways of differential metabolites in rose cultivars, providing a data reference and ideas for the study of secondary metabolites in roses.

## Data availability statement

The original contributions presented in the study are included in the article/**Supplementary Material**. Further inquiries can be directed to the corresponding authors.

## Author contributions

HZ designed the experiment. WQ, JJ, and CQ performed the experiments, among which WQ and JJ performed most of the experiments and analyzed the data. WQ and JJ wrote the original draft. CL and HZ revised the manuscript. All authors contributed to the article and approved the submitted version.
