# Supplementary material for: Characterization of volatiles in flowers from four Rosa chinensis cultivars by HS-SPME-GC × GC-QTOFMS
Source: Front Plant Sci. 2023 May 8;14:1060747. doi: 10.3389/fpls.2023.1060747 (PMC10211245; doi:10.3389/fpls.2023.1060747)
Supplement: Supplementary file 1 [file DataSheet_1.pdf]

Table S1. Identification of volatile constituents in *Rosa* samples.

No.	Compounds	$^{1}tR^{a}$ (min)	$^{2}tR^{b}$ (sec)	CAS	Chemical formula	DMF[c]	RMF [d]	RI_{exp}[e]	RI_{lit}[f]
1	Acetoin	4.09	1.44	513-86-0	$C_4H_8O_2$	848	902	-	690
2	6-Methyl-3,4-dihydro-2H-pyran	4.78	1.47	16015-11-5	$C_6H_{10}O$	899	841	-	-
3	1,3-Butanediol	4.99	2.07	107-88-0	$C_4H_{10}O_2$	804	807	-	805
4	Hexanal	5.19	1.71	66-25-1	$C_6H_{12}O$	883	932	-	776
5	2-Hexenal	6.29	2.25	505-57-7	$C_6H_{10}O$	850	863	-	835
6	(*Z*)-3-Hexen-1-ol	6.49	1.88	928-96-1	$C_6H_{12}O$	811	818	-	839
7	1-Hexanol	6.58	1.82	111-27-3	$C_6H_{14}O$	827	828	-	854
8	Styrene	7.18	2.47	100-42-5	C_8H_8	809	935	-	878
9	3-Methylcyclopentyl acetate	7.28	2.08	24070-70-0	$C_8H_{14}O_2$	789	873	901	-
10	Hexanoic acid, methyl ester	7.88	2.08	106-70-7	$C_7H_{14}O_2$	858	871	924	907
11	(*E*)-3-Hexenoic acid, methyl ester	8.09	2.37	13894-61-6	$C_7H_{12}O_2$	879	853	932	920
12	Benzaldehyde	8.98	3.99	100-52-7	C_7H_6O	887	888	967	933
13	(*E*)-2-Hexenoic acid, methyl ester	8.99	2.59	13894-63-8	$C_7H_{12}O_2$	858	836	966	948
14	Hexanal dimethyl acetal	9.29	1.88	1599-47-9	$C_8H_{18}O_2$	814	932	977	964
15	Alpha-Myrcene	9.38	1.81	1686-30-2	$C_{10}H_{16}$	841	845	981	-
16	6-Methyl-5-hepten-2-one	9.58	2.66	110-93-0	$C_8H_{14}O$	834	867	989	964
17	S-sulcatol	9.68	2.26	1569-60-4	$C_8H_{16}O$	855	816	993	974
18	beta-Myrcene	9.68	1.85	123-35-3	$C_{10}H_{16}$	839	847	993	983
19	alpha-Methoxytoluene	9.69	3.16	538-86-3	$C_8H_{10}O$	886	901	994	973
20	(*E,Z*)-2,4-Heptadienal	9.99	3.13	4315-03-5	$C_7H_{10}O$	786	819	1005	982
21	alpha-Phellandrene	10.08	2.05	99-83-2	$C_{10}H_{16}$	845	866	1008	998
22	(*Z*)-hex-4-enyl acetate	10.09	2.44	42125-17-7	$C_8H_{14}O_2$	892	911	1008	1000
23	*E*-2-hexenyl acetate	10.29	2.51	2497-18-9	$C_8H_{14}O_2$	874	924	1015	995
24	(+)-4-Carene	10.38	2.07	29050-33-7	$C_{10}H_{16}$	870	879	1018	998
25	2-methyl-3-methylidenecyclopentane-1-carbaldehyde	10.39	3.20	826337-64-8	$C_8H_{12}O$	785	788	1019	-
26	*p*-Cymene	10.69	2.40	99-87-6	$C_{10}H_{14}$	832	865	1029	1014
27	*D*-Limonene	10.79	2.09	138-86-3	$C_{10}H_{16}$	837	878	1033	1023
28	Benzyl alcohol	10.98	4.21	100-51-6	C_7H_8O	871	878	1041	1012
29	Benzeneacetaldehyde	11.18	4.60	122-78-1	C_8H_8O	871	884	1048	1011
30	trans-Ocimen	11.29	2.05	13877-91-3	$C_{10}H_{16}$	852	865	1050	1038
31	Gamma-terpinene	11.58	2.27	99-85-4	$C_{10}H_{16}$	869	883	1061	1050
32	Linalool oxide	11.99	2.50	5989-33-3	$C_{10}H_{18}O_2$	850	856	1076	1064
33	(*E,E*)-3,5-Octadien-2-one	11.99	3.32	30086-02-3	$C_8H_{12}O$	819	873	1076	1068
34	4-Isopropenyltoluene	12.48	3.00	1195-32-0	$C_{10}H_{12}$	837	876	1094	1074
35	trans-Linalool oxide	12.49	2.60	34995-77-2	$C_{10}H_{18}O_2$	827	847	1094	1074
36	Terpinolene	12.49	2.34	586-62-9	$C_{10}H_{16}$	877	883	1093	1079
37	Linalool	12.68	2.44	78-70-6	$C_{10}H_{18}O$	863	864	1101	1086
38	Nonanal	12.88	2.48	124-19-6	$C_9H_{18}O$	866	867	1108	1083
39	trans-Rose oxide	13.08	2.49	5258-11-7	$C_{10}H_{18}O$	872	916	1115	1115
40	Phenylethyl alcohol	13.28	4.41	60-12-8	$C_8H_{10}O$	898	901	1120	1088
41	Octanoic acid, methyl ester	13.38	2.35	111-11-5	$C_9H_{18}O_2$	780	892	1126	1108

42	Rose oxide	13.59	2.61	16409-43-1	$C_{10}H_{18}O$	795	887	1133	1102
43	(*4E,6Z*)-allo-Ocimene	13.59	2.28	7216-56-0	$C_{10}H_{16}$	820	829	1133	1131
44	(-)-trans-Pinocarveol	13.59	3.12	547-61-5	$C_{10}H_{16}O$	857	820	1133	1126
45	*p*-Mentha-1,5,8-triene	13.69	2.53	21195-59-5	$C_{10}H_{14}$	856	819	1136	1104
46	1,4-Dimethoxybenzene	14.09	4.83	150-78-7	$C_{8}H_{10}O_{2}$	772	784	1152	1132
47	Isopinocarveol	14.28	3.22	6712-79-4	$C_{10}H_{16}O$	855	792	1158	1176
48	Dill ether	14.39	3.16	74410-10-9	$C_{10}H_{16}O$	815	876	1162	1171
49	2-Nonenal	14.48	2.88	18829-56-6	$C_{9}H_{16}O$	837	878	1165	1135
50	Benzyl acetate	14.69	3.24	140-11-4	$C_{9}H_{10}O_{2}$	887	887	1173	1151
51	trans-Linalool 3,7-oxide	14.89	3.18	39028-58-5	$C_{10}H_{18}O_{2}$	821	851	1180	1164
52	Isoneral	15.08	3.04	72203-97-5	$C_{10}H_{16}O$	830	864	1187	1151
53	Alpha-Terpineol	15.38	3.17	98-55-5	$C_{10}H_{18}O$	850	861	1197	1175
54	Decanal	15.68	2.57	112-31-2	$C_{10}H_{20}O$	857	859	1208	1185
55	Cumaldehyde	15.68	3.08	122-03-2	$C_{10}H_{12}O$	801	875	1208	1215
56	Benzylacetone	15.69	3.12	2550-26-7	$C_{10}H_{12}O$	843	824	1208	1217
57	7-methyl-3-methylene-6-Octen-1-ol	16.09	2.93	13066-51-8	$C_{10}H_{18}O$	843	881	1222	-
58	Eucarvone	16.18	3.89	503-93-5	$C_{10}H_{14}O$	782	834	1226	-
59	Nonanoic acid, methyl ester	16.18	2.40	1731-84-6	$C_{10}H_{20}O_{2}$	835	848	1226	1208
60	Levoverbenone	16.19	3.68	1196-01-6	$C_{10}H_{14}O$	850	888	1226	1197
61	Alpha-Citronellol	16.29	2.66	6812-78-8	$C_{10}H_{20}O$	838	850	1229	1212
62	cis-Geraniol	16.38	3.01	106-25-2	$C_{10}H_{18}O$	869	869	1233	1213
63	Citronellol	16.38	2.65	106-22-9	$C_{10}H_{20}O$	854	856	1233	1211
64	Nerol	16.39	2.91	106-25-2	$C_{10}H_{18}O$	862	862	1233	1213
65	cis-Citral	16.68	3.38	5392-40-5	$C_{10}H_{16}O$	828	855	1244	1241
66	Neral	16.69	3.41	106-26-3	$C_{10}H_{16}O$	877	879	1244	1218
67	Isogeraniol	16.78	3.03	5944-20-7	$C_{10}H_{18}O$	840	855	1247	1237
68	1,3-Di-tert-butylbenzene	17.08	2.39	1014-60-4	$C_{14}H_{22}$	898	922	1258	1247
69	Geraniol	17.08	2.99	106-24-1	$C_{10}H_{18}O$	843	843	1258	1237
70	2-Phenylethyl acetate	17.19	4.26	103-45-7	$C_{10}H_{12}O_{2}$	899	907	1262	1228
71	Pulegone	17.28	3.66	89-82-7	$C_{10}H_{16}O$	854	766	1266	1249
72	alpha-Citral	17.48	3.47	141-27-5	$C_{10}H_{16}O$	867	867	1273	1249
73	3,5-Dimethoxytoluene	17.49	4.33	4179-19-5	$C_{9}H_{12}O_{2}$	906	929	1273	1256
74	2-Phenyl-2-butenal	17.69	5.23	4411-89-6	$C_{10}H_{10}O$	896	908	1281	1266
75	Geranyl formate	18.38	2.85	105-86-2	$C_{11}H_{18}O_{2}$	843	847	1305	1282
76	Theaspirane	18.39	2.76	36431-72-8	$C_{13}H_{22}O$	857	870	1305	1288
77	Methyl geranoate	18.99	3.00	1189-09-9	$C_{11}H_{18}O_{2}$	857	894	1328	1302
78	Decanoic acid, methyl ester	18.99	2.36	110-42-9	$C_{11}H_{22}O_{2}$	853	881	1327	1308
79	Citronellol acetate	19.68	2.53	150-84-5	$C_{12}H_{20}O_{2}$	871	872	1354	1335
80	alpha-Cubebene	19.69	2.20	17699-14-8	$C_{15}H_{24}$	874	893	1354	1351
81	Nerol acetate	19.98	2.86	141-12-8	$C_{12}H_{20}O_{2}$	882	884	1366	1343
82	Megastigma-4,6(*Z*),8(*E*)-triene	19.09	2.75	51468-85-0	$C_{13}H_{20}$	826	847	1332	1340
83	3-Methoxyphenethyl alcohol	20.19	5.33	5020-41-7	$C_{9}H_{12}O_{2}$	820	860	1376	1345
84	(*E*)-2-Butyl-2-octenal	20.28	2.54	13019-16-4	$C_{12}H_{22}O$	838	901	1378	1363
85	1,3-Pentanediol,2,2,4-trimethyl-,1-isobutyrate	20.29	2.95	77-68-9	$C_{12}H_{24}O_{3}$	772	879	1378	1365
86	alpha-Copaene	20.39	2.36	3856-25-5	$C_{15}H_{24}$	875	879	1381	1376

87	Geranyl acetate	20.48	2.92	105-87-3	$C_{12}H_{20}O_2$	878	883	1386	1361
88	beta-Cubebene	20.79	2.44	13744-15-5	$C_{15}H_{24}$	844	877	1397	1385
89	Ethyl geranate	20.79	2.97	32659-21-5	$C_{12}H_{20}O_2$	811	852	1397	-
90	Isoeugenol	20.99	4.64	5912-86-7	$C_{10}H_{12}O_2$	897	903	1406	1381
91	Methyleugenol	21.08	4.54	93-15-2	$C_{11}H_{14}O_2$	871	889	1410	1372
92	1,3,5-Trimethoxybenzene	21.18	5.36	621-23-8	$C_9H_{12}O_3$	870	874	1415	1385
93	alpha-Gurjunene	21.29	2.53	489-40-7	$C_{15}H_{24}$	812	838	1417	1406
94	beta-Caryophyllene	21.58	2.68	87-44-5	$C_{15}H_{24}$	890	892	1430	1419
95	beta-Copaene	21.79	2.61	18252-44-3	$C_{15}H_{24}$	881	882	1438	1426
96	Tetrahydroionone	22.09	3.05	60761-23-1	$C_{13}H_{24}O$	801	813	1451	1427
97	(+)-epi-Bicyclosesquiphellandrene	22.19	2.73	54274-73-6	$C_{15}H_{24}$	851	858	1455	-
98	Geranylacetone	22.28	3.02	3796-70-1	$C_{13}H_{22}O$	868	881	1459	1429
99	Calarene	22.59	2.80	17334-55-3	$C_{15}H_{24}$	841	859	1472	1450
100	gamma-Muurolene	22.99	2.72	30021-74-0	$C_{15}H_{24}$	881	883	1488	1472
101	Germacrene D	23.09	2.87	23986-74-5	$C_{15}H_{24}$	874	885	1493	1477
102	beta-Ionone	23.09	3.58	79-77-6	$C_{13}H_{20}O$	874	874	1493	1466
103	Isohomogenol	23.49	4.78	93-16-3	$C_{11}H_{14}O_2$	835	887	1511	1487
104	alpha-Muurolene	23.49	2.81	10208-80-7	$C_{15}H_{24}$	871	872	1509	1494
105	Butylated Hydroxytoluene	23.79	3.14	128-37-0	$C_{15}H_{24}O$	862	863	1522	1494
106	gama-Cadinene	23.89	2.94	39029-41-9	$C_{15}H_{24}$	869	870	1526	1507
107	Methyl laurate	23.99	2.38	111-82-0	$C_{13}H_{26}O_2$	859	862	1530	1508
108	Calamenene	24.08	3.33	483-77-2	$C_{15}H_{22}$	882	889	1535	1510
109	beta-Cadinene	24.09	2.87	523-47-7	$C_{15}H_{24}$	846	846	1534	1522
110	alpha-Cubebene	24.29	3.03	29837-12-5	$C_{15}H_{24}$	840	898	1543	1525
111	alpha-Cadinene	24.39	2.97	24406-05-1	$C_{15}H_{24}$	818	892	1547	1528
112	alpha-Calacorene	24.59	3.61	21391-99-1	$C_{15}H_{20}$	885	906	1556	1531
113	alpha-Corocalene	26.39	3.98	20129-39-9	$C_{15}H_{20}$	850	868	1632	1607
114	gama-Eudesmol	26.58	3.60	1209-71-8	$C_{15}H_{26}O$	875	878	1641	1618
115	Cedrelanol	26.79	3.60	5397-11-1	$C_{15}H_{26}O$	858	873	1649	1627
116	alpha-Cadinol	26.89	3.53	481-34-5	$C_{15}H_{26}O$	818	826	1654	1642
117	beta-Eudesmol	27.08	3.81	473-15-4	$C_{15}H_{26}O$	857	864	1662	1636
118	alpha-Eudesmol	27.18	3.66	473-16-5	$C_{15}H_{26}O$	878	887	1667	1643
119	Cadalene	27.59	4.38	483-78-3	$C_{15}H_{18}$	894	894	1685	1654
120	Methyl tetradecanoate	28.59	2.39	124-10-7	$C_{15}H_{30}O_2$	830	850	1729	1708
121	Isobutyl phthalate	31.59	4.47	84-69-5	$C_{16}H_{22}O_4$	879	883	1874	1850
122	Dibutyl phthalate	33.48	4.73	84-74-2	$C_{16}H_{22}O_4$	899	901	1971	1924

NOTE:
[a] ^{1}tR: retention times of 1D.
[b] ^{2}tR: retention times of 2D.
[c] DMF: direct match factor–similarity of the deconvoluted mass spectra of unknowns to those in the NIST mass spectral library.
[d] RMF: reverse match factor–a reverse search matching factor between the library and deconvoluted mass spectra of unknown.
[e] RI_{exp}: experimental retention index.
[f] RI_{lit}: retention index of the literature.
