# Supplementary material for: Characterization of volatiles in flowers from four Rosa chinensis cultivars by HS-SPME-GC × GC-QTOFMS
Source: Front Plant Sci. 2023 May 8;14:1060747. doi: 10.3389/fpls.2023.1060747 (PMC10211245; doi:10.3389/fpls.2023.1060747)
Supplement: Supplementary file 2 [file DataSheet_2.pdf]

Table S2. The classification, KEGG pathway, relative content and aroma description of volatile compounds in *Rosa* samples

No.	Compounds	Class [a]	cpd_ID [b]	kegg_ko [c]	Relative content [d]				Aroma description	Aroma references [e]
					RCG	RBR	RPP	RF		
1	Acetoin	Ketones	C00466	--	6.48±0.24a	0.00b	0.00b	0.00b	green, berry	Web
2	6-Methyl-3,4-dihydro-2H-pyran	Others	--	--	8.4±3.43a	8.70±1.50a	0.00b	0.00b	--	--
3	1,3-Butanediol	Alcohols	C20335	--	27.17±14.12a	0.00b	0.00b	0.00b	odorless	Web
4	Hexanal	Aldehydes	C02373	ko00140	48.12±6.75b	109.3±7.44a	35.47±7.45bc	25.58±2.11c	grassy, green	Feng et al., 2019
5	2-Hexenal	Aldehydes	C08497	--	21.8±5.47c	129.09±21.97a	97.81±9.11ab	115.1±2.16a	green, fruity	Feng et al., 2019
6	(*Z*)-3-Hexen-1-ol	Alcohols	C08492	ko00592,ko01110	0.00c	0.00c	9.85±1.9a	2.62±1.61b	fresh, green	Web
7	1-Hexanol	Alcohols	C00854	ko00930,ko01120,ko01220	2.68±0.09b	5.9±0.93a	0.00c	0.00c	resin, flower, green	Zhu et al., 2016
8	Styrene	Aromatic hydrocarbons	C19506	--	0.00b	0.00b	1.49±0.07a	0.00b	sweet, floral	Web
9	3-Methylcyclopentyl acetate	Esters	--	--	0.00b	12.49±4.18a	0.00b	0.00b	--	--
10	Hexanoic acid, methyl ester	Esters	--	--	0.00c	2.64±0.51a	0.45±0.13b	3.77±0.56a	fruity	Web
11	(*E*)-3-Hexenoic acid, methyl ester	Esters	--	--	0.00c	0.00c	3.05±1.42b	23.1±3.02a	sweet, green, fruity	Web
12	Benzaldehyde	Aldehydes	C00261	ko00623,ko00627,ko01100,ko01120,ko01220	88.96±12.66a	18.3±3.94b	13.74±1.36b	18.51±4.05b	fruity, green	Web
13	(*E*)-2-Hexenoic acid, methyl ester	Esters	--	--	0.00b	0.00b	0.00b	2.21±0.61a	fruity, green	Web
14	Hexanal dimethyl acetal	Aldehydes	--	--	0.00b	0.00b	1.09±0.34a	0.85±0.47a	green sweet	Web
15	alpha-Myrcene	Alkenes	C06074	ko00902,ko01062,ko01110	0.00b	6.71±2.25a	0.00b	0.00b	--	--
16	6-Methyl-5-hepten-2-one	Ketones	C07287	--	7.37±1.62a	11.56±1.59a	3.72±1.06b	9.28±0.92a	fruity, green	Feng et al., 2018
17	S-sulcatol	Alcohols	--	--	0.00b	10.63±1.28a	0.00b	0.00b	weet oily green	Web
18	beta-Myrcene	Alkenes	C06074	ko00902,ko01062,ko01110	64.38±8.03b	115.62±17.97a	33.91±7.85c	19.77±7.27c	fruity, clove-like	An et al., 2019
19	alpha-Methoxytoluene	Aromatic hydrocarbons	--	--	0.00b	0.00b	0.18±0.06a	0.00b	fruity	Web
20	(*E,Z*)-2,4-Heptadienal	Aldehydes	--	--	0.00b	0.00b	0.00b	2.58±0.46a	fatty, green	Feng et al., 2019
21	alpha-Phellandrene	Alkenes	--	--	9.85±2.62ab	24.14±8.52a	4.2±2.33b	0.5±0.08c	grassy, green	Feng et al., 2018
22	(*Z*)-hex-4-enyl acetate	Esters	--	--	0.00b	0.00b	11.52±4.73a	13.4±5.88a	--	--
23	*E*-2-hexenyl acetate	Esters	--	--	0.00b	0.00b	4.64±1.27a	0.00b	sweet, privet, green	Web
24	(+)-4-Carene	Alkenes	--	--	3.61±0.58b	30.62±11.32a	0.00c	0.00c	green, sweet	Web
25	2-methyl-3-methylidenecyclopentane-1-carbaldehyde	Aldehydes	--	--	9.15±2.00b	11.72±4.00ab	10.91±4.64ab	20.57±3.06a	--	--
26	*p*-Cymene	Aromatic hydrocarbons	C06575	ko00622,ko01100,ko01120,ko01220	6.01±1.38ab	13.82±4.97a	2.16±0.72b	1.55±1.00b	floral, grassy	Feng et al., 2018
27	*D*-Limonene	Alkenes	C06099	ko00902,ko00903,ko01062,ko01100,ko01110	55.11±10.75b	115.91±27.52a	31.71±9.97c	11.92±2.68d	fresh, citrusy, sweet	Feng et al., 2019
28	Benzyl alcohol	Alcohols	C03485	--	214.8±24.68a	1.00±0.22c	45.67±4.18b	47.53±10.58b	sweet, flower	Zhu et al., 2016
29	Benzeneacetaldehyde	Aldehydes	C00601	ko00360,ko00643,ko01100,ko01120	103.37±27.16a	76.06±26.92ab	55.36±2.71b	50.27±2.17b	sweet, floral	Web
30	trans-Ocimen	Alkenes	C09873	--	34.04±5.27b	72.6±17.38a	19.73±5.74c	10.14±5.02c	floral, green	An et al., 2019
31	Gamma-terpinene	Alkenes	C09900	--	4.4±0.81a	12.5±4.99a	0.00c	1.13±0.82b	turpentine, woody	An et al., 2019
32	Linalool oxide	Alcohols	--	--	5.55±4.28a	0.00b	7.25±3.21a	0.00b	floral, sweet	Web
33	(*E,E*)-3,5-Octadien-2-one	Ketones	--	--	0.00b	0.00b	0.00b	3.12±0.57a	fruity, green	Web
34	4-Isopropenyltoluene	Aromatic hydrocarbons	--	--	0.00b	0.00b	1.95±0.30a	0.00b	phenolic, spicy	Web
35	trans-Linalool oxide	Alcohols	--	--	14.72±2.54b	39.98±10.65a	6.49±3.6c	0.00d	flower, lavender	Zhu et al., 2016
36	Terpinolene	Alkenes	C06075	--	0.00d	17.96±8.07a	4.19±1.67b	0.63±0.12c	pine, forest	Amanpour et al., 2019
37	Linalool	Alcohols	C05853	ko00360	0.00c	54.3±16.61a	23.77±7.98b	0.00c	floral, woody, citrusy	Amanpour et al., 2019; Feng et al., 2019
38	Nonanal	Aldehydes	--	--	32.29±3.31a	0.00c	0.00c	10.45±1.13b	fatty, citrus, green	Zhu et al., 2016
39	trans-Rose oxide	Alkenes	--	--	11.57±3.91a	0.00b	0.00b	0.00b	floral	Feng et al., 2018
40	Phenylethyl alcohol	Alcohols	--	--	265.63±29.24a	310.56±3.22a	3.7±1.29b	6.99±2.36b	red rose, green	Qin et al., 2013
41	Octanoic acid, methyl ester	Esters	--	--	0.00b	0.00b	10.74±4.57a	22.06±9.66a	sweet, green	Web
42	Rose oxide	Alkenes	--	--	7.02±3.61a	0.00b	0.00b	0.00b	red rose, green	Web
43	beta-Ocimene	Alkenes	--	--	21.74±3.08a	32.97±7.91a	6.86±2.65b	0.00c		
44	(-)-trans-Pinocarveol	Alcohols	--	--	0.00c	2.99±0.79a	1.09±0.08b	0.00c	warm woody	Web
45	p-Mentha-1,5,8-triene	Alkenes	--	--	0.00c	14.34±3.01a	2.03±0.52b	0.00c	roasted	Web
46	1,4-Dimethoxybenzene	Aromatic hydrocarbons	--	--	14.38±4.94a	0.00b	0.00b	0.00b	sweet, green	Web
47	Isopinocarveol	Alcohols	--	--	0.00b	1.99±0.28a	0.00b	0.00b	woody	Web
48	Dill ether	Alkenes	--	--	0.00b	0.00b	1.36±0.13a	1.99±0.39a	green	Web
49	2-Nonenal	Aldehydes	--	--	0.00c	14.82±3.23a	0.39±0.02b	0.00c	green, cucumber	Web
50	Benzyl acetate	Esters	C15513	--	2.95±0.19a	0.00b	3.19±0.56a	0.00b	sweet floral	Web
51	trans-Linalool 3,7-oxide	Alcohols	--	--	5.46±0.95b	16.09±2.79a	2.07±1.00b	0.00c	woody	Web
52	Isoneral	Aldehydes	--	--	6.42±2.23ab	10.96±4.14a	2.41±0.80b	0.00c	green	Web
53	Alpha-Terpineol	Alcohols	C09902	--	1.04±0.33b	9.2±4.59a	1.96±1.02ab	0.27±0.13c	pine, terpene	Web
54	Decanal	Aldehydes	C12307	--	5.16±0.78b	9.2±1.31a	3.31±0.76b	4.78±0.68b	sweet, floral	Feng et al., 2019
55	Cumaldehyde	Aldehydes	C06577	ko00622,ko01100,ko01120,ko01220	0.00b	7.56±1.52a	0.00b	0.00b	spicy, cumin, green	Web
56	Benzylacetone	Ketones	--	--	3.32±0.12a	0.00b	0.00b	0.00b	floral	Web
57	7-methyl-3-methylene-6-	Alcohols	--	--	14.27±5.49a	9.44±4.63a	0.00b	0.00b	--	--
58	Eucarvone	Ketones	--	--	0.00b	6.31±2.39a	0.00b	0.00b	minty	Web
59	Nonanoic acid, methyl ester	Esters	--	--	0.00b	0.00b	6.79±3.27a	4.2±1.24a	sweet, fruity	Web
60	Levoverbenone	Ketones	--	--	4.24±1.43a		1.17±0.32b	6.01±0.26a	minty, fruity	Qin et al., 2013
61	Alpha-Citronellol	Alcohols	C09849	ko00281,ko01062,ko01110	0.00b	3.67±0.74a	0.00b	0.00b	floral, red rose	Web
62	cis-Geraniol	Alcohols	C09871	ko00281,ko01062	0.00b	47.92±15.69a	0.00b	0.00b	sweet	Web
63	Citronellol	Alcohols	C09849	ko00281,ko01062,ko01110	158.68±16.08a	77.65±5.27b	11.84±4.38c	5.52±2.07c	floral, rose	Feng et al., 2018; An et al., 2019
64	Nerol	Alcohols	C09871	ko00281,ko01062	0.00b	0.00b	35.76±3.71a	26.16±12.50a	floral, sweet	Feng et al., 2018
65	cis-Citral	Aldehydes	C09847	ko00281,ko01062,ko01110	0.00c	42.95±11.68a	9.86±3.01b	10.84±5.69b	lemon, sweet	Web
66	Neral	Aldehydes	C01499	ko00281,ko01062,ko01110	35.05±11.33a	0.00c	0.00c	7.77±4.83b	sweet, citral	Web
67	Isogeraniol	Alcohols	C09871	ko00281,ko01062	1.51±0.08a	4.2±2.49a	0.00b	0.00b	floral, sweet	An et al., 2019
68	1,3-Di-tert-butylbenzene	Aromatic hydrocarbons	--	--	6.41±3.44b	6.81±1.83b	11.1±1.4a	11.89±0.83a	--	--
69	Geraniol	Alcohols	C09871	ko00281,ko01062	24.99±17.96b	93.22±8.54a	40.87±2.98b	33.95±5.80b	rose-like, floral, sweet	Qin et al., 2013; Feng et al., 2018
70	2-Phenylethyl acetate	Esters	C12303	--	54.15±24.49a	18.02±5.87ab	1.32±0.21b	0.00c	floral, rose	Web
71	Pulegone	Ketones	--	--	0.00b	8.15±2.87a	0.00b	0.00b	peppermint, fresh	Web
72	alpha-Citral	Aldehydes	C01499	ko00281,ko01062,ko01110	36.05±9.88ab	54.62±11.3a	15.66±3.83b	0.00c	lemon-like	An et al., 2018
73	3,5-Dimethoxytoluene	Aromatic hydrocarbons	--	--	34.07±4.07b	51.35±17.85a	7.42±2.57c	65.22±7.66a	-	

74	2-Phenyl-2-butenal	Aldehydes	--	--	9.97±2.00a	0.00b	0.00b	0.00b	sweet	Web
75	Geranyl formate	Esters	--	--	1.69±0.68a	2.33±0.29a	0.00b	0.00b	fresh, rose	Web
76	Theaspirane	Others	--	--	0.00b	0.00b	0.00b	5.41±1.39a	tea herbal	Web
77	Methyl geranoate	Esters	--	--	13.9±5.35a	9.77±3.72a	7.05±0.82a	2.25±0.73b	green	Web
78	Decanoic acid, methyl ester	Esters	--	--	4.59±1.17a	0.00b	3.97±0.62a	4.71±2.44a	fruity, floral	Web
79	Citronellol acetate	Esters	--	--	1.8±0.94b	10.22±4.28a	3.5±1.82ab	0.00c	floral, green, rose	Web
80	alpha-Cubebene	Alkenes	C09647	--	13.6±4.71a	0.00b	5.02±1.51a	0.00b	herbal	Web
81	Nerol acetate	Esters	C09861	--	0.00c	8.03±4.32a	4.44±1.86a	0.94±0.09b	floral, rose	Web
82	Megastigma-4,6(*Z*),8(*E*)-	Alkenes	--	--	0.00b	0.00b	0.00b	3.8±1.68a	--	--
83	3-Methoxyphenethyl alcohol	Alcohols	--	--	2.86±0.28a	0.00b	0.00b	0.00b	--	--
84	(*E*)-2-Butyl-2-octenal	Aldehydes	--	--	0.00c	6.16±1.1a	0.00c	0.7±0.2b	--	--
85	1,3-Pentanediol,2,2,4-trimethyl-,1-isobutyrate	Esters	--	--	26.66±1.83a	0.00c	0.00c	3.41±2.28b	--	--
86	alpha-Copaene	Alkenes	C09639	--	7.04±4.61a	0.00b	17.14±4.86a	0.00b	woody	Web
87	Geranyl acetate	Esters	C09861	--	1.86±0.76b	15.92±8.46a	11.45±2.8a	0.00c	floral, rose	Web
88	beta-Cubebene	Alkenes	C09648	--	5.93±2.29a	0.00b	0.00b	0.00b	citrus, fruity	Web
89	Ethyl geranate	Esters	--	--	1.93±0.34a	0.00b	0.00b	0.00b	woody, rose	Web
90	Isoeugenol	Alcohols	C10469	ko00940	1.81±0.4a	0.00b	0.00b	0.00b	clove-like, smoke	Amanpour et al., 2019
91	Methyleugenol	Alcohols	C10454	ko00940	52.87±12.15a	4.95±0.89c	0.00d	17.81±2.99b	sweet, fresh	--
92	1,3,5-Trimethoxybenzene	Aromatic hydrocarbons	--	--	64.74±6.56a	6.73±1.14c	6.62±1.98c	26.6±8.37b	--	--
93	alpha-Gurjunene	Alkenes	--	--	4.68±1.36a	0.00b	2.07±0.67a	0.00b	woody	Web
94	beta-Caryophyllene	Alkenes	C09629	ko00909,ko01100,ko01110	17.49±2.58a	5.19±0.72b	0.00c	7.58±2.85ab	sweet, woody	Web
95	beta-Copaene	Alkenes	--	--	10.28±1.29a	0.00b	6.65±2.10a	0.00b	--	--
96	Tetrahydroionone	Ketones	--	--	0.00b	0.00b	0.00b	13.82±4.67a	--	--
97	(+)-epi-	Alkenes	--	--	0.00b	0.00b	5.97±2.63a	0.00b	--	--
98	Geranylacetone	Ketones	C13297	--	0.00b	2.17±0.41a	1.48±0.66a	0.00b	green	Song et al., 2019
99	Calarene	Alkenes	--	--	9.08±2.44a	0.00b	0.00b	0.00b	radiant, woody	Web
100	gamma-Muurolene	Alkenes	--	--	11.21±2.71a	4.53±1.34b	12.48±4.09a	0.00c	herbal	Web
101	Germacrene D	Alkenes	--	--	0.00b	0.00b	8.55±2.92a	0.00b	woody	Web
102	beta-Ionone	Ketones	--	--	0.00b	0.00b	0.00b	9.19±2.87a	violet, flower	Zhu et al., 2016
103	Isohomogenol	Alcohols	--	--	7.14±1.05a	0.00b	0.00b	0.00b	spicy, clove	Web
104	alpha-Muurolene	Alkenes	--	--	19.75±3.9a	5.23±1.42b	15.98±5.23a	0.23±0.06c	woody	Web
105	Butylated Hydroxytoluene	Aromatic hydrocarbons	C14693	--	9.21±0.27a	10.16±2.24a	5.17±0.87b	6.92±0.72b	mild	
106	gama-Cadinene	Alkenes	--	--	0.00b	0.00b	14.78±4.82a	0.00b	spicy, woody	Qin et al., 2013
107	Methyl laurate	Esters	--	--	4.4±0.14ab	2.93±0.9b	0.00c	5.11±1.13a	waxy, soapy	Web
108	Calamenene	Aromatic hydrocarbons	--	--	5.47±1.83a	2.59±0.67a	0.00b	0.00b	herb	The Good Scents Company Information
109	beta-Cadinene	Alkenes	C09625	--	32.17±7.05a	4.02±0.91b	14.71±2.77a	0.97±0.13c	spicy, woody	Qin et al., 2013
110	alpha-Cubebene	Alkenes	C09647	--	5.79±1.56a	0.00b	0.00b	0.00b	spicy fruity	Web
111	alpha-Cadinene	Alkenes	C16815	--	0.00b	0.00b	6.97±2.67a	0.00b	spicy, woody	Qin et al., 2013
112	alpha-Calacorene	Aromatic hydrocarbons	--	--	5.17±1.21a	0.00b	5.06±1.65a	0.00b	woody	Web
113	alpha-Corocalene	Alkenes	--	--	1.98±0.47a	0.00b	1.71±0.6a	0.00b	--	--
114	gama-Eudesmol	Alcohols	--	--	0.00b	6.47±2.29a	0.00b	0.00b	sweet	Web
115	Cedrelanol	Alcohols	--	--	0.00c	0.00c	2.58±0.89a	0.43±0.04b	--	--
116	alpha-Cadinol	Alcohols	--	--	5.15±0.94a	0.00b	0.00b	0.00b	herb	Web
117	beta-Eudesmol	Alcohols	--	--	3.13±1.05a	5.17±2.26a	0.00b	0.00b	woody	Web
118	alpha-Eudesmol	Alcohols	--	--	1.93±0.53a	2.57±0.98a	0.00b	0.00b	--	--
119	Cadalene	Aromatic hydrocarbons	--	--	2.24±0.33a	0.00b	1.97±0.31a	0.00b	--	--
120	Methyl tetradecanoate	Esters	--	--	1.75±0.72a	0.00b	0.00b	1.83±0.55a	fatty	Web
121	Isobutyl phthalate	Esters	C15205	--	2.38±0.39a	2.05±0.91a	0.00b	0.00b	--	--
122	Dibutyl phthalate	Esters	C14214	--	4.81±0.21a	4.47±0.43a	3.34±0.31b	3.08±0.39b	faint odor	Web

NOTE:

[a] Method of classification based on literature. https://doi.org/10.1016/j.indcrop.2020.112818

[b] KEGG ID: Kyoto Encyclopedia of Genes and Genomes, https://www.metaboanalyst.ca/MetaboAnalyst/upload/ConvertView.xhtml

[c] KO (KEGG Orthology) database is a database of molecular functions represented in terms of functional orthologs.https://www.genome.jp/kegg-bin/get_htext?ko00001

[d] Relative content: results are the means of three repetitions as μg/g, Data indicate mean ± standard error. Different lowercase letters indicate significant differences at $p < 0.05$.

[e] Aroma description of compounds retrieved from The Good Scent Company: www.the good scent company.com

-- not detected
