# Supplementary material for: Characterization of volatiles in flowers from four Rosa chinensis cultivars by HS-SPME-GC × GC-QTOFMS
Source: Front Plant Sci. 2023 May 8;14:1060747. doi: 10.3389/fpls.2023.1060747 (PMC10211245; doi:10.3389/fpls.2023.1060747)
Supplement: Supplementary file 3 [file DataSheet_3.docx]

**
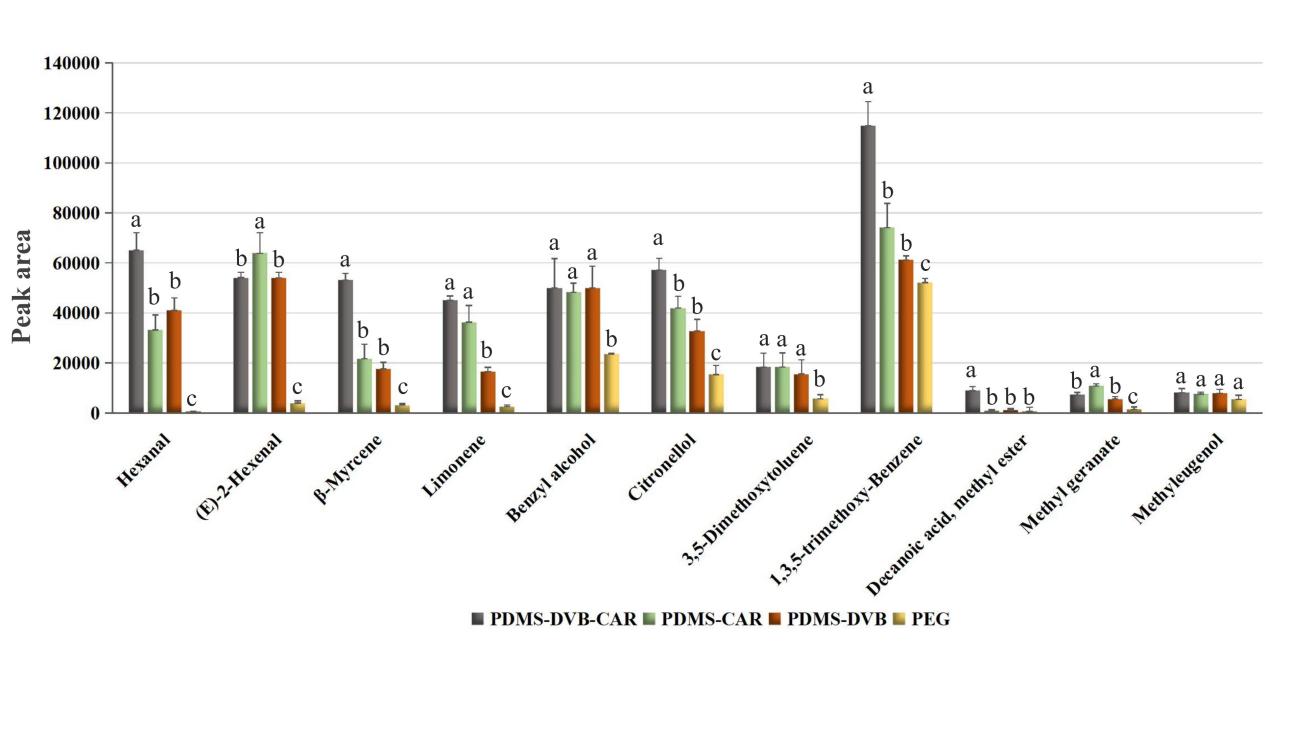
Figure S1. Effect of the type of coating on the extraction efficiency of the selected compounds obtained by HS-SPME-GC×GC/QTOF-MS analysis.**



**Figure S2. Representative 2-D GC contour plot of two volatiles in four cultivars.**
